# A Case of a Penetrating Traumatic Head Injury Due to a Ceiling Fan

**DOI:** 10.7759/cureus.26350

**Published:** 2022-06-26

**Authors:** Yoon N Ong

**Affiliations:** 1 Pediatric Emergency Department, Hospital Tuanku Azizah, Kuala Lumpur, MYS

**Keywords:** extra-axial hematoma, depressed skull fracture, compression bandage, penetrating head injury, ceiling fan

## Abstract

A ceiling fan is a hidden enemy on the ceiling of a house. When the blunt blades rotate at a high speed, it has the capability to penetrate the skull bones. This case highlights the uncommon presentation of fan blade injury and the importance of compression bandage.

## Introduction

A ceiling fan is a piece of important household equipment in Malaysia due to its hot climate. However, the recklessness of parents leaving their children unattended while playing at home can lead to serious penetrating traumatic head injury. The severity of the head injury depends on the materials that are used to make the fan blade and the speed of the fan. Even though minor injuries such as lacerations on the scalp and face are commonly reported, depressed skull fractures and intracranial bleed have been reported as well [1,2].

## Case presentation

A previously healthy seven-year-old Malay boy presented to the Pediatric Emergency Department of Hospital Tuanku Azizah with a deep laceration wound over the right frontal region of the scalp after his head was struck by a ceiling fan. The boy was playing at home and had climbed onto a cupboard, which led to the incident. On examination, he was alert, pink, and in pain. Vital signs showed a heart rate of 120 beats per minute, blood pressure of 134/84 mmHg, respiratory rate of 25 breaths per minute, oxygen saturation of 99% on air, and temperature of 37°C. Examination of the head revealed a semilunar-shaped deep laceration wound measuring 6 cm long, 1 cm wide, and 2 cm deep, exposing the frontal bone with a visible fracture line. There was blood oozing continuously from the wound. Compression bandage was applied on the wound and a primary survey was conducted. The primary and secondary survey findings were normal. The case was referred promptly to the neurosurgery team. Intranasal fentanyl was given for pain relief. Laboratory investigations, full blood count, and coagulation profile were normal.

**Figure 1:**
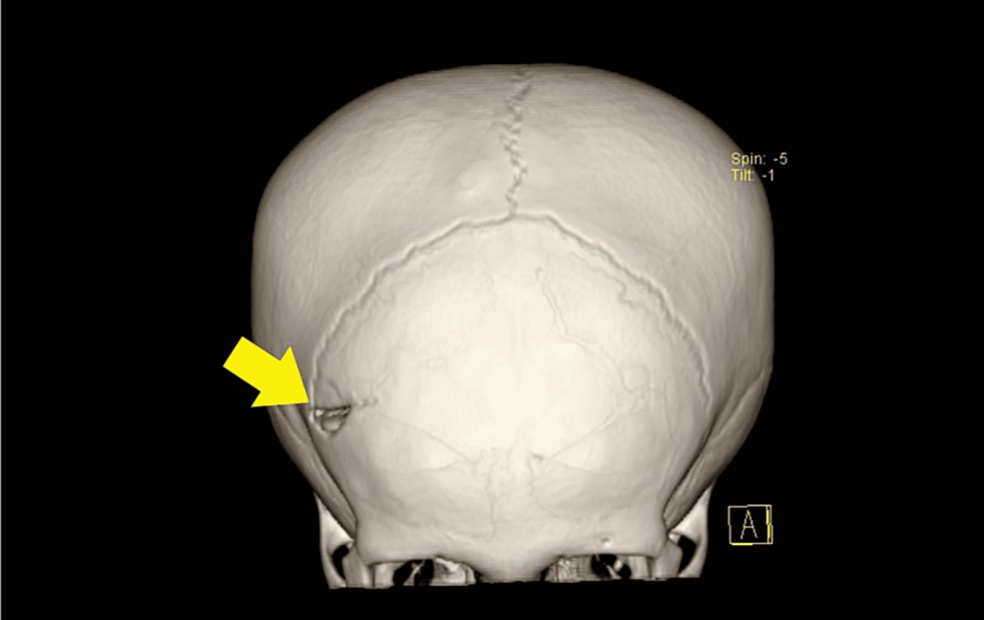
The yellow arrow shows the semilunar-shaped skull fracture at the right frontal bone in the three-dimensional-reconstructed computed tomography of the brain.

**Figure 2:**
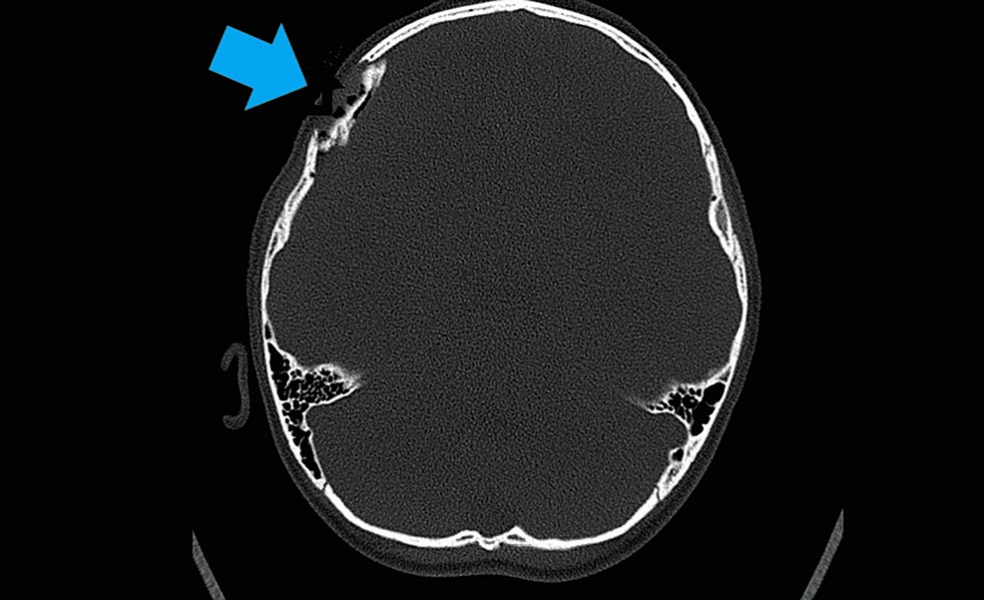
The blue arrow shows the right depressed frontal bone fracture with pneumocranium on the non-contrast computed tomography of the brain (bone window).

**Figure 3:**
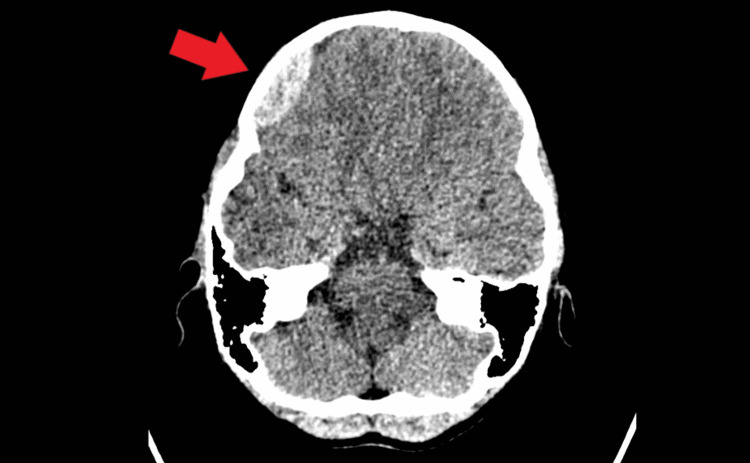
The red arrow shows a convex-shaped hyperdensity over the right frontal region indicating a right extra-axial hematoma on a non-contrast computed tomography of the brain.

Subsequently, computed tomography of the brain (Figure 1-3) was done which showed a depressed right frontoparietal bone fracture with right frontal extra-axial hematoma. An emergency right craniotomy, evacuation of the clot, and elevation of the depressed skull fracture were done five hours after the incident. The procedure was uneventful and the boy was discharged 48 hours after the operation with a neurosurgery follow-up.

## Discussion

Penetrating traumatic brain injury is rare in Malaysia. Based on a retrospective observational study by Rohana et al. in 1998, only 0.06% of penetrating traumatic brain injury cases presented to the emergency department of Hospital Kuala Lumpur Malaysia from 1993 to 1994 [3]. In a retrospective cohort study done by the neurosurgical unit of Hospital Kuala Lumpur Malaysia, 14 cases of penetrating traumatic head injuries due to ceiling fans were reported from 2000 to 2002. Out of the 14 cases, four needed neurosurgery intervention for depressed skull fracture [1]. Meanwhile, in Australia, a cohort study reported 136 presentations to an emergency department in Townsville from 2005 to 2010. In their study, the authors found seven patients with skull fractures, and only one of them required neurosurgical intervention [2]. The most commonly reported mechanisms of injury in these studies were jumping from bunk beds, jumping from furniture, and being lifted by adults [1,2]. The boy in this case was playing on top of the cupboard when his frontal forehead was hit by the blade of the ceiling fan. He suffered a depressed skull fracture with an extra-axial hematoma needing neurosurgery intervention.

The application of an effective compression bandage was important in this case to prevent progression to hemorrhagic shock due to the bleeding wound. The wound was initially compressed using a flat square gauze with fingers providing pressure on the bleeding point. Then, the flat square gauze was replaced by a folded nugget of gauze and subsequently bigger pieces of gauze on top. An elastic bandage was then applied to the forehead to secure the gauze in its position. This technique was described by Shokrollahi et al. in 2006 as a temporary measure to control hemorrhage on limbs [4]. The physiology behind this method is based on the equation: pressure = force/area. This method successfully controlled the bleeding from the wound in this case.

Parents have the paramount duty to ensure that home is a safe place for children physically and emotionally. Prevention of injuries caused by ceiling fans is always better than treating the injuries. Australian Competition and Consumer Commission came out with safety advice for the usage of ceiling fans. According to the safety advice, ceiling fans need to be placed at least 2.1 m from the floor and bunk beds and furniture at least 2 m away from the ceiling fans, and no lifting of children when underneath or near a ceiling fan [5]. However, such safety measures are lacking in Malaysia.

## Conclusions

Ceiling fans have the potential to cause penetrating traumatic head injuries even though they are rare. The boy in our case suffered a depressed skull fracture with extra-axial hematoma which needed neurosurgical intervention in contrast to most cases in previous studies which only required toilet and suturing of the laceration wound. This case serves as a reminder of the importance of awareness regarding the safety measures of having a ceiling fan at home. Control of bleeding which is the initial step in the primary survey of a trauma patient plays an important role in preventing hemorrhagic shock. Therefore, all physicians working in pediatric emergency departments should have the necessary knowledge and skills for controlling hemorrhage.
